# Malignant glomus tumor of the lung with multiple metastasis: a rare case report

**DOI:** 10.1186/s12957-014-0423-3

**Published:** 2015-02-07

**Authors:** Saibin Wang, Chuanbiao Ding, Junwei Tu

**Affiliations:** Department of Pneumology, Zhejiang University Jinhua Hospital, Jinhua Municipal Central Hospital, 321000 Jinhua, China; Department of Internal Medicine, Jinhua Municipal Central Hospital Pan-an Branch, 1# Luoshan Road, Pan-an, 322300 China

**Keywords:** Glomus tumor, Lung, Metastasis

## Abstract

Malignant glomus tumor, or glomangiosarcoma, is a very rare mesenchymal neoplasm that, when seen, occurs in visceral organs. Despite having histologic features of malignancy, these tumors usually do not metastasize. However, when metastasis occurs, this disease is often fatal. Our report presents the case of a 59-year-old female patient with a highly aggressive and widely metastatic glomus tumor of the lung.

## Background

Glomus tumors are uncommon benign neoplasms that customarily originate from glomus bodies in the dermis or subcutis of the extremities. Extracutaneous glomus tumors occur but are very rare, especially in visceral organs such as the stomach, mediastinum, trachea, lung, ileum, and kidney [1-5]. A few cases of glomangiosarcoma in visceral organs have been reported; nevertheless, these all involved local invasion and metastasis occurred scarcely [6]. Herein, we report a case with a highly aggressive and widely metastatic malignant glomus tumor of the lung.

## Case presentation

**Figure 1:**
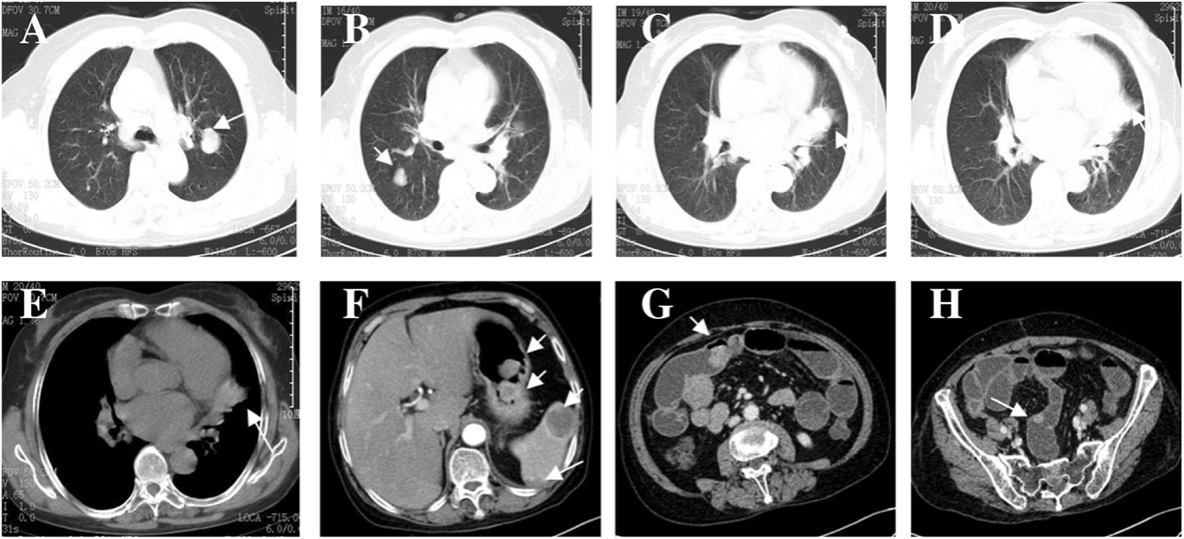
**CT scans. (A, B)** multiple mass in bilateral lungs; **(C–E)** the lobulated glomangiosarcoma was abutting the left cardiac border in the left inferior lobe; **(F–H)** multiple metastasis: to stomach and spleen **(F)**, to jejunum **(G)**, to ileum **(H)** (arrows).

**Figure 2:**
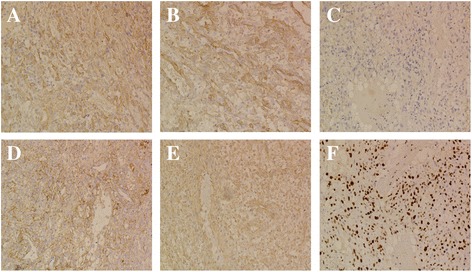
**Immunohistochemical staining.** SMA was expressed strongly by tumor cells from lung **(A)** and from stomach **(B)**; type IV collagen expressed weakly by tumor cells from lung **(C)** and strongly from stomach **(D)**; vimentin positive expression by tumor cells from lung **(E)**; Ki-67 positive expression is about 60% by tumor cells from lung **(F)**.

A 59-year-old female patient (living in eastern China, at an altitude of 550 m) presented to our hospital complaining of an ongoing cough and hemoptysis for 4 months and melena for 1 week. The patient’s medical history was unremarkable. The auscultation revealed attenuated respiratory sound in bilateral lungs and no rales or wheezing were noted. There was no superficial node enlargement. Laboratory investigations showed normocytic anemia (hemoglobin level 58 g/L) and a platelet count of 294 × 10^9^ /L. The computed tomography (CT) scan of the chest showed multiple masses in bilateral lungs with the largest measuring 25 mm in diameter in the left upper lobe (Figure 1A,B) and an irregularly protruding edge, lobulated mass was abutting the left cardiac border in the left inferior lobe (Figure 1D–E). Also noted were mildly enlarged mediastinal nodes. Enhanced CT scans of the abdomen demonstrated gastrointestinal lumen multiple clumps, with the upper right jejunum intussusception, incomplete obstruction by tumor, and multiple mass in spleen and ileum considering tumor metastasis (Figure 1F–H). To make a definite diagnosis of tumor, an endoscopic study was performed. Bronchoscopy revealed new features in the left lung lingual lobe cavity, the lumen was completely blocked. Gastroscopy revealed stomach multiple clumps. Pathology and immunohistochemistry were subsequently performed. Immunohistochemically, tumor cells from the lung were positive for SMA (Figure 2A), CD163, F8, vimentin (Figure 2E), and collagen IV (Figure 2C), whereas they were negative for CD21, CD31, CD34, CD35, CD117, Bcl-2, CK7, CKlow, EMA, HMB45, desmin, myosin, s-100, and TTF-1 (Table 1). Tumor cells from stomach were positive for SMA (Figure 2B) and collagen IV (Figure 2D), and F8 was rare weakly positive, whereas they were negative for CD31, CD117, CK, dog-1, HMB45, desmin, and s-100. Ki-67 labeling index reached about 60% (Figure 2F) from lung specimen and about 40% from stomach (Table 1). The histopathological findings were consistent with malignant glomus tumor [7]. With deteriorating medical conditions and widespread metastasis, the patient was submitted to a palliative therapy. Bronchial artery embolization was given for hemostasis. The patient developed more generalized weakness and eventually died within 20 weeks of diagnosis.

**Table 1:** **Immunohistochemical characteristics of the tumor cells**

**Antibodies to**	**Tumor cells from lung**	**Tumor cells from stomach**
EMA	Negative	Not done
SMA	Positive	Positive
Actin	Rare weakly positive	Not done
Vimentin	Positive	Not done
CD a1	Negative	Not done
CD 21	Negative	Not done
CD 31	Negative	Negative
CD 34	Negative	Not done
CD 35	Negative	Not done
CD 99	Rare weakly positive	Not done
CD 117	Negative	Negative
CD 163	Positive	Not done
F8	Positive	Rare weakly positive
Dog-1	Not done	Negative
HMB 45	Negative	Negative
S-100	Negative	Negative
Desmin	Negative	Negative
Collagen IV	Rare weakly positive	Positive
Bcl-2	Negative	Not done
CK	Not done	Negative
CK 7	Negative	Not done
CK low	Negative	Not done
Myosin	Negative	Not done
TTF-1	Negative	Not done
Ki-67 labeling index	About 60%	About 40%

## Discussion

Glomus tumors typically present as solitary or multicentric lesions in the dermis or subcutis, with characteristic subungual location. Extracutaneous presentations occur but are rare, especially in the visceral organs, where glomus bodies are sparse or even absent, including the respiratory tract, gastrointestinal tract, and mediastinum. Within the respiratory tract, the trachea is the most frequent site of involvement. Primary glomus tumors of the lung, especially with metastasis, are extremely rare. To the best of our knowledge, only three cases have been reported in the English literature. The first case of a glomangiomyoma of the lung with widespread metastasis was reported by Gaertner et al. [8] in 2000, in which the patient developed widespread metastatic disease to the lungs, upper mediastinum, brain, liver, and subcutaneous tissue of the lower limb after surgery, then she received chemotherapy and ultimately died 68 weeks after surgery. A decade later, Liu et al. [9] reported another case of primary malignant glomus tumor in the lung with multiple metastasis to the left lung, visceral pleura, adjacent bronchi, and the vessel walls. The patient also underwent surgery and chemotherapy, but died 4 days after surgery due to pulmonary failure. This was followed by Hohenforst-Schmidt et al. [10], who reported a case of glomus tumor in the left hilum of the lung, but multiple lung metastasis were evident at the 6-year follow-up visit; the patient was submitted to a pneumonectomy of the left lung and was disease free within in the surgical limits.

**Figure 3:**
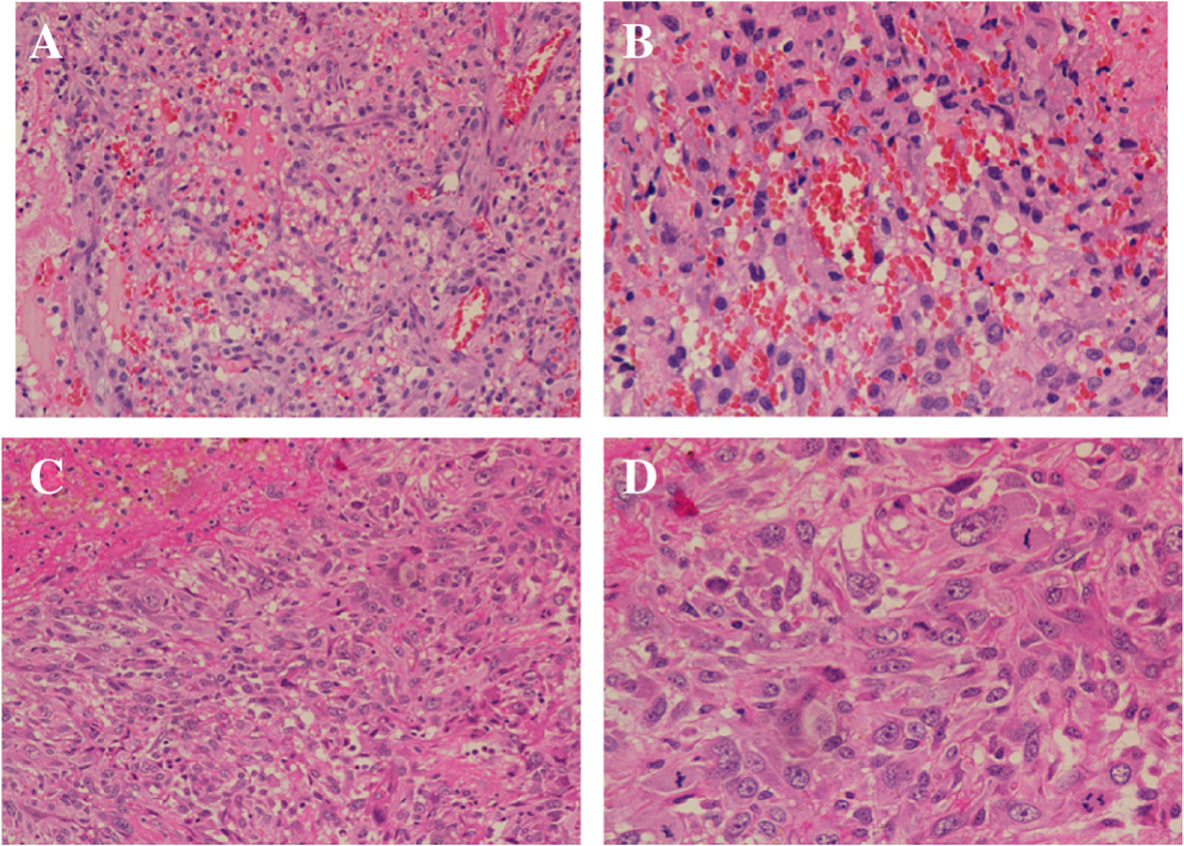
**Microscopic appearance (H&E).** Histocytologic features of glomangiosarcoma from lung (**A**, ×200) and from stomach (**C**, ×200). Cytological atypia and increased mitotic activity was observed from lung (**B**, ×400) and from stomach (**D**, ×400).

Folpe et al. [7] proposed the following criteria for malignant glomus tumor: tumors with a deep location and a size of more than 20 mm, or atypical mitotic figures, or moderate to high nuclear grade and ≥5 mitotic figures per 50 high power fields. In our case, the diameter of the largest mass was 25 mm in the left upper lobe (Figure 1A). Microscopically, nuclear atypia of tumor cells with high mitotic activity was evident (Figure 3), fulfilling the criteria for malignancy. Given the rarity of reported cases of malignant glomus tumor of the lung, these patients can be misdiagnosed easily with other pulmonary disorders. Malignant glomus tumors have to be differentiated from other lesions such as Ewing’s tumors/primitive neurectodermal tumors, carcinoid tumors, neuroendocrine carcinoma, sclerosing hemangioma, malignant melanoma, synovial sarcoma, malignant mesothelioma, hemangiopericytoma, and metastatic tumors. Histologic and immunohistochemical features are fundamental in establishing the diagnosis of malignant glomus tumors, the clinicopathologic features and immunohistochemical profiles of which were summarized initially by Khoury et al. [11]. In this case, it was slightly difficult to differentiate from a multiple gastrointestinal glomus tumor with lung metastasis; however, the possibility of metastatic malignant glomus tumors from other organs to the lung was obviated in light of the clinical and radiological evidence. The patient was referred to hospital due to long term pulmonary symptoms rather than gastrointestinal discomfort. However, and even more importantly, the irregularly protruding edge, lobulated mass in the left inferior lobe (Figure 1D–E), considered a primary lesion, did not meet the characteristics of metastasis in radiology.

Surgical resection is curative and remains the mainstay of treatment in reported cases of malignant glomus tumor diagnosed with or without local metastasis [2], and long term follow-up is greatly recommended. Milia et al. [12] reported a successful outcome with combined radiotherapy and chemotherapy in a case of malignant glomus tumor in a 40-year-old man presenting with a lesion in the upper cervical region. In our case, the patient lost the opportunity for surgery given the widespread metastasis and did not undergo radiotherapy or chemotherapy because of asthenia, resulting in fatality.

## Conclusions

Malignant glomus tumor of the lung, particularly with multiple metastasis, is exceedingly rare. The cornerstone of diagnosis is pathological features and immunostaining. This case, combined with the other three cases mentioned above (Table 2), indicate that a case of malignant glomus tumor with widespread metastasis is often fatal. Diagnosis before metastasis and effortlessly aggressive surgical treatment is essential of excellent prognosis in this disease.

**Table 2:** **Cases of primary pulmonary glomus tumors with metastasis**

**Case**	**Sex /Age(years)**	**Symptoms**	**Site**	**Metastatic sites**	**Treatment**	**Follow-up**
Gaertner et al. [8]	M/69	Hemoptysis	RUL	Lungs, mediastinum, brain, liver, lower limb	Lobectomy, chemotherapy	DOD at 68 weeks
Liu et al. [9]	M/48	Fever, dry cough, hemoptysis	LUL, LHL	Left lung, visceral pleura, the vessel walls, bronchi	Lobectomy, chemotherapy	DOD at 4 days
Hohenforst-Schmidt et al. [10]	F/35	Thoracic pain	LHL	Left lung	Lobectomy	FOD in surgical limits, N/A
Wang et al.	F/59	Cough, hemoptysis, melena	LIL	Lungs, stomach, jejunum, ileum, mediastinal nodes	Palliative therapy	DOD at 20 weeks

RUL, Right upper lobe; LUL, Left upper lobe; LIL, Left inferior lobe; LHL, Left hilum of the lung; FOD, Free of disease; N/A, Not available.

## Consent

Written informed consent was obtained for publication of this report and accompanying images. A copy of the written consent is available for review by the Editor-in-Chief of this journal.
